# The role of hypoxia related hormones responses in acute mountain sickness susceptibility individuals unaccustomed to high altitude

**DOI:** 10.1371/journal.pone.0292173

**Published:** 2023-10-05

**Authors:** Bayan Fayazi, Vahid Tadibi, Kamal Ranjbar

**Affiliations:** 1 Department of Exercise Physiology, Faculty of Sport Sciences, Razi University, Kermanshah, Iran; 2 Department of Exercise Physiology, Faculty of Sport Sciences, Bandar Abbas Branch, Islamic Azad University, Bandar Abbas, Iran; Vanderbilt University Medical Center, UNITED STATES

## Abstract

Acute mountain sickness (AMS) is caused by rapid ascent to altitude (>2500 m) and remains a poorly understood pathophysiological condition. Accordingly, we investigated the relationship between acute exposure to high altitude and hypoxia related biochemical proteins. 21 healthy subjects (Female (8) and male (13), Age: 36.7±8.5, BMI: 23.2±3.1) volunteers participated in this project and fasting blood samples were taken before (sea level) and after 1 and 24-h exposure to high altitude (3,550 m). Blood oxygen saturation (SpO_2_), AMS status (Lake Louise Score) and serum HIF-1, Endothelin-1, VEGF and Orexin-A were measured (via ELISA) at 1, 6 and 24 h after exposure to high altitude. Pre-ascent measurement of hypoxia related proteins (Orexin-A, HIF-1, VEGF and Endothelin-1) where all significantly (<0.05) higher in the AMS-resistant individuals (No-AMS) when compared to AMS susceptible individuals (AMS+). Upon ascent to high altitude, 11 out of 21 volunteers had AMS (10.1±0.6 in AMS+ vs. 0.9±0.6 in No-AMS, P<0.05) and presented with lower resting SpO_2_ levels (77.7±0.4 vs. 83.5±0.3 respectively, p<0.05). Orexin-A, HIF-1, VEGF and Endothelin-1, significantly increased 24 hrs after exposure to high altitude in both AMS+ and No-AMS. The response of Orexin-A was similar between two groups, also, HIF-1 elevation 24 hrs after exposure to altitude was more in AMS+ (13% vs. 19%), but the increase of VEGF and Endothelin-1, 1 and 24 hrs after exposure to altitude in No-AMS was double that of AMS+. Hypoxia related proteins include Orexin-A, HIF-1, VEGF and Endothelin-1 may play a pathophysiological role in those who are susceptible to AMS.

## Introduction

It is well known that acute mountain sickness (AMS) caused by rapid ascent above 2500 m is the most common physiological dysfunction that occurs at high altitude [1]. Typically, AMS is usually recognized with a combination several symptoms, such as headache, gastrointestinal symptoms, sleep disturbance, general weakens, insomnia, and loss of appetite. Symptoms begin two to three hours after ascent and, if unresolved or untreated, may develop to high altitude pulmonary edema (HAPE) and high altitude cerebral edema (HACE). Some people are more susceptible to AMS than others [2]; therefore, awareness of the individual susceptibility for AMS would be a useful tool to prevent or limit subsequent harm. An exact strategy to predict AMS susceptibility remains elusive [3].

Although the underlying etiology of AMS remains obscure, it is at least in part related to alterations in cellular milieu. For example, previous studies showed that Hypoxia inducible factor-1 (HIF-1) pathway have a pivotal role in altitude diseases, especial those with excessive erythrocytosis [4]. HIF-1 is a main transcription factors in hypoxia condition that acts as a key regulator of oxygen homeostasis [5], including the activation of vascular endothelial growth factor (VEGF) as a key factor in vascular permeability and basement membrane damage. Related, Ding et al showed that VEGF is an important component of the pathogenesis of AMS [5]. Also, increase of circulating Endothelin-1 during ascent to high altitude, may play pathological roles in AMS and HAPE [6]. It has been noted that Endothelin-1 is a potent vasoconstrictor and increase vascular permeability in hypoxia [7]. Finally, Orexin-A—mainly produced by hypothalamic neurons—play crucial roles in the control of feeding, sleep–wake cycle, metabolic rate and pathogenesis of narcolepsy [8]. It has been shown that confirmed that hypoxia exposure decrease Orexin-A gene expression in the piglet hypothalamus [9] and this change may be related to the hypoxia-induced behavioral deficit such as narcolepsy and anorexia at high altitude. Experiments confirmed that VEGF, Orexin-A and Endothelin-1 are some of the main target genes of HIF-1 [10]. However, the relationship between these factors and AMS still remains to be elucidated. Given that the pathophysiology of AMS remains nebulous, we aimed to compare changes—and potential relationships—between hypoxia related proteins levels between AMS-susceptible and AMS-resistant individuals.

## Material and methods

### Participants

21 healthy subjects (Female (8) and male (13), Age: 36.7±8.5, BMI: 23.2±3.1) voluntary participated in this study. All subjects were fit, nonsmokers, with no history of cardiopulmonary and the subjects were not allowed to take any medication. All participants were long-term lowland residents (1190 m) and had not been exposed to altitudes above 2500 m within 30 days ago. All participants gave written informed consent to participate and the protocol of this study was approved by Razi university ethics Committee (KUMS.REC.1396.255) and all experiments were performed in accordance with relevant guidelines and regulations.

### Study design

This was an observational, prospective cohort study. Eligible participants recruited through advertisement. Inclusion criteria included young people (20–40 years old) lowland residents with 20<BMI<25. Exclusion criteria included chronic and acute illness, heavy activity or high-altitude (>2500 m) exposure history in the past month and recent drug use.

#### Sea-level data collection

The examination lasted two days. 24 h before exposure to high altitude the main physiological parameters, including fasting blood samples, heart rate, and SpO_2_ were measured at rest (1190 m above sea level at 9 A.M). All volunteers had a standardized diet throughout the study.

#### Altitude data collection

Following 24 hrs, volunteers rapidly ascended from 1,190 to 3,550 m (Tochal Mountain located in Central Alborz mountain range in northern Iran, Mount Damavand, the highest mountain in Iran measuring 5,610.0 m (18,405.5 ft), is located in the Central Alborz Mountains) within 30 min by gondola and remained at this elevation for a further 24 h. Blood samples were taken at 1 and 24 h after exposure to high altitude in a semi-recumbent position from an antecubital vein. Serum was allowed to clot for 30 min before centrifugal separation and storage at−80°C until batch analyses of HIF-1, Endothelin-1, VEGF and Orexin-A.

Oximax handheld pulse oximeter (Onyx II 9550; Nonin, Minnesota) was used for SpO_2_ measurement. SpO_2_ was measured every 1 h during exposure to high altitude. Also, AMS was assessment by Lake Louise Acute Mountain Sickness Scoring System [11]. Subjects were classified as suffering from severe AMS and NO-AMS. Those with severe AMS trekkers were considered to be susceptible to AMS when their Lake Louise score (LLS) was ≥4. AMS was evaluated in 1, 6 and 24 hrs after exposure to high altitude.

Serum HIF-1 concentrations were measured using commercially available ELISA kit: (Human Hypoxia-Inducible Factor 1 (HIF-1), ELISA, ZellBio GmbH, Ulm, Germany, Intraassay CV%: 2 and Sensitivity: 0.1 ng/ml), serum VEGF concentrations were measured using commercially available ELISA kit: (Human Vascular Endothelial cell Growth Factor (VEGF), ELISA, ZellBio GmbH, Ulm, Germany, Intraassay CV%: 4.3 and Sensitivity: 10 ng/L), serum Endothelin-1 concentrations were measured using commercially available ELISA kit: (Human Endothelin 1, ELISA, ZellBio GmbH, Ulm, Germany, Intraassay CV%: 5.3 and Sensitivity: 1 ng/L) and serum Orexin-A concentrations were measured using commercially available ELISA kit: (Human Orexin A, ELISA, ZellBio GmbH, Ulm, Germany, Intraassay CV%: 3.7 and Sensitivity: 2.5 pg/ml).

### Statistical analyses

Statistical analyses were performed with SPSS 23.0 (IBM Corporation, Armonk, New York, USA). Normality was determined via the Shapiro-wilk test. Comparison of means was performed using independent samples t test (normally distributed variables) and Mann-Whitney test (for no normally distributed variables) for participants without AMS (No-AMS) as compared to participants with AMS (AMS+). Also, in order to compare means within two groups unpaired t test was used. A 2×3 mixed-plot factorial repeated measures ANOVA was performed to analyze for comparisons of variable changes between the two groups from sea level to 1 h and 24 h after exposure to high altitude. Pearson correlations test was used to determine the relationship between the study variables. Significance was set as p≤0.05. Values are given as Mean±SD.

## Results

Of the 21 participants recruited at the beginning of the study, 11 (52%) individuals were AMS+ while the others were No-AMS (The average of AMS in three time point: 10.10±0.55 in AMS+ vs. 0.9±0.59 in No-AMS). Difficulty sleeping and headache were the most frequent symptoms of AMS (72% and 66%, respectively), followed by gastrointestinal symptoms (34%), fatigue (20%) and dizziness (13%). Lake Louise score in 1, 6 and 24 hrs after exposure to high altitude was shown in Table 1. It should to be noted that 2 subjects with AMS, several hours after exposure to hypoxia, due to AMS severity transferred to the sea level.

**Table 1:** Lake Louise score after exposure to high altitude (3550 m).

After high altitude exposure	AMS+	No-AMS
**1 h**	9.09±0.653*	0.30±0.153
**6 hrs**	13.09±0.513*†	1.60±0.306†
**24 hrs**	8.11±0.512*	0.80±0.133

* Significant difference between AMS and No-AMS groups

† Significant difference compared 1 and 24 hrs after high altitude exposure. Sig≤0.05, values are Mean±SD.

Also, the general characteristic of the volunteers in the two groups are presented in Table 2.

**Table 2:** Baseline characteristics of the subjects.

*Variable*	*AMS+ (n = 11)*	No-AMS *(n = 10)*
*Sex*	Female (4)/male(7)	Female (4)/male(6)
*Age*, *years*	36.91±1.69	36.50±1.58
*Body weight*, *kg*	70.55±3.95	67.65±2.21
*Body Height*, *m*	1.75 ±0.30	1.72±0.28
*BMI*, *kg/m*^*2*^	23.1±1.4	23.4±1.09

Values are Mean±SD.

### Sea level

The level of Orexin-A in No-AMS group was 1256±482 pg/ml and 432.8±51.0 pg/ml in AMS+ group (T = -5.6, P = 0.0001). Moreover, HIF-1 level in No-AMS group was higher than AMS+ group (14.2±7.9 ng/ml vs. 2.7±0.3 ng/ml; T = -4.8, P = 0.0001). Similarly, both serum VEGF and Endothelin-1 was higher in the No-AMS group when compared to the AMS+ group (all P<0.01). Finally, SpO_2_ was comparable between groups (T = 0.5, P = 0.6).

### Exposure to Hypoxia (1 and 24 h after exposure to high altitude)

#### Orexin-A

**Fig 1:**
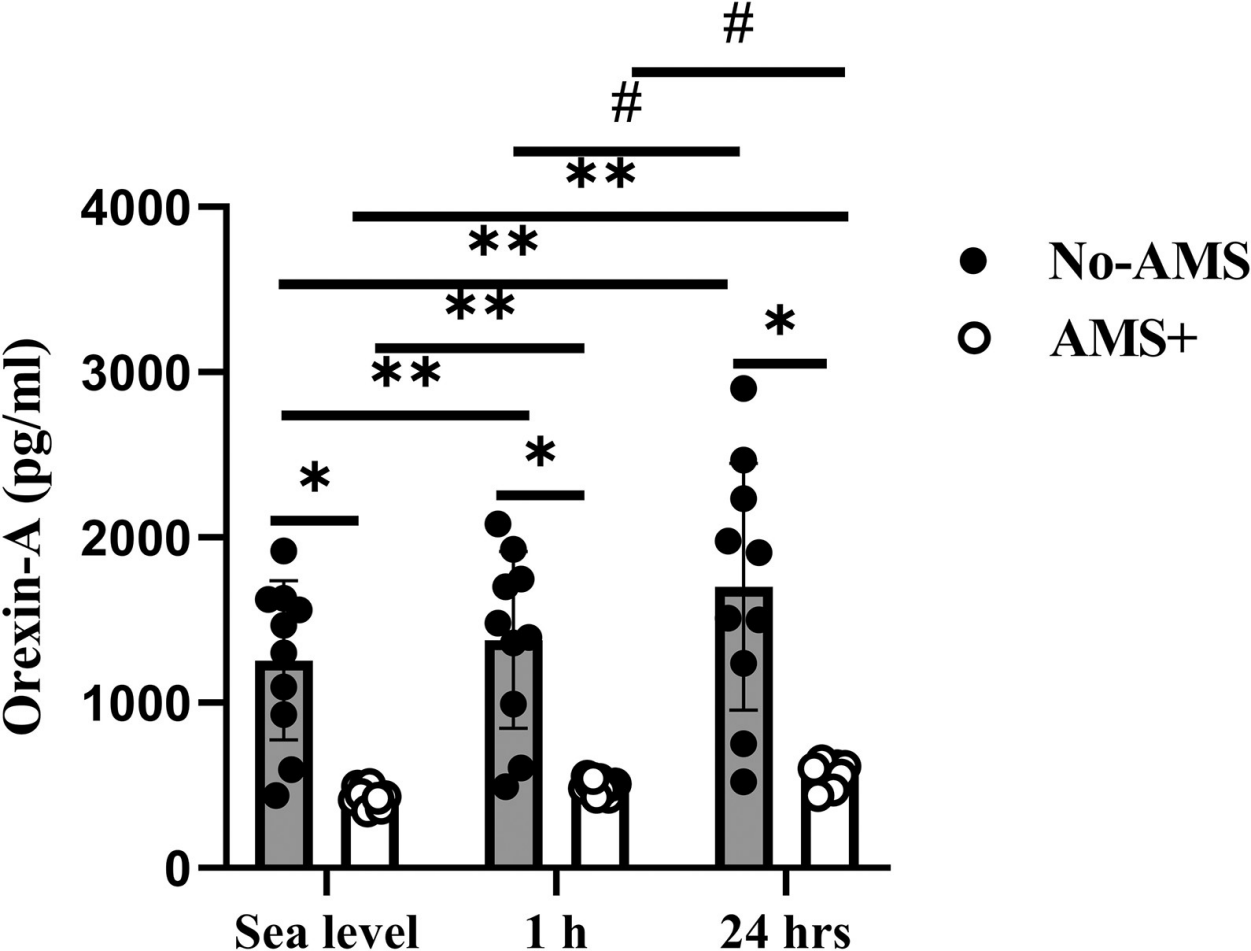
Orexin-A response to exposure to high altitude in No-AMS and AMS+ groups. * Significant difference between AMS and No-AMS groups, ** Significant different vs. Sea level, **#** Significant different vs. 1 h after exposure to high altitude. Sig≤0.05, values are Mean±SD.

In No-AMS group, Orexin-A level increased 10% and 35% at 1 and 24 h after exposure to high altitude (respectively, P = 0.01 and P = 0.001) in comparison to sea level (Fig 1). The level of Orexin-A at 24 hrs after exposure to high altitude was more than 1 h after exposure to high altitude (P = 0.001).

In AMS+ group, circulating Orexin-A rose 15% (P = 0.01) and 36% (P = 0.0001) in 1 and 24 h after exposure to altitude in comparison to sea level. Serum Orexin-A was more in 24 h after exposure to altitude compared to 1 h after exposure to altitude (P = 0.0001).

Also, serum Orexin-A was significantly different between AMS+ and No-AMS at the 1 and 24 h after exposure to high altitude (respectively, p = 0.0001 and p = 0.0001).

#### Hypoxia Inducible Factor-1 (HIF-1)

**Fig 2:**
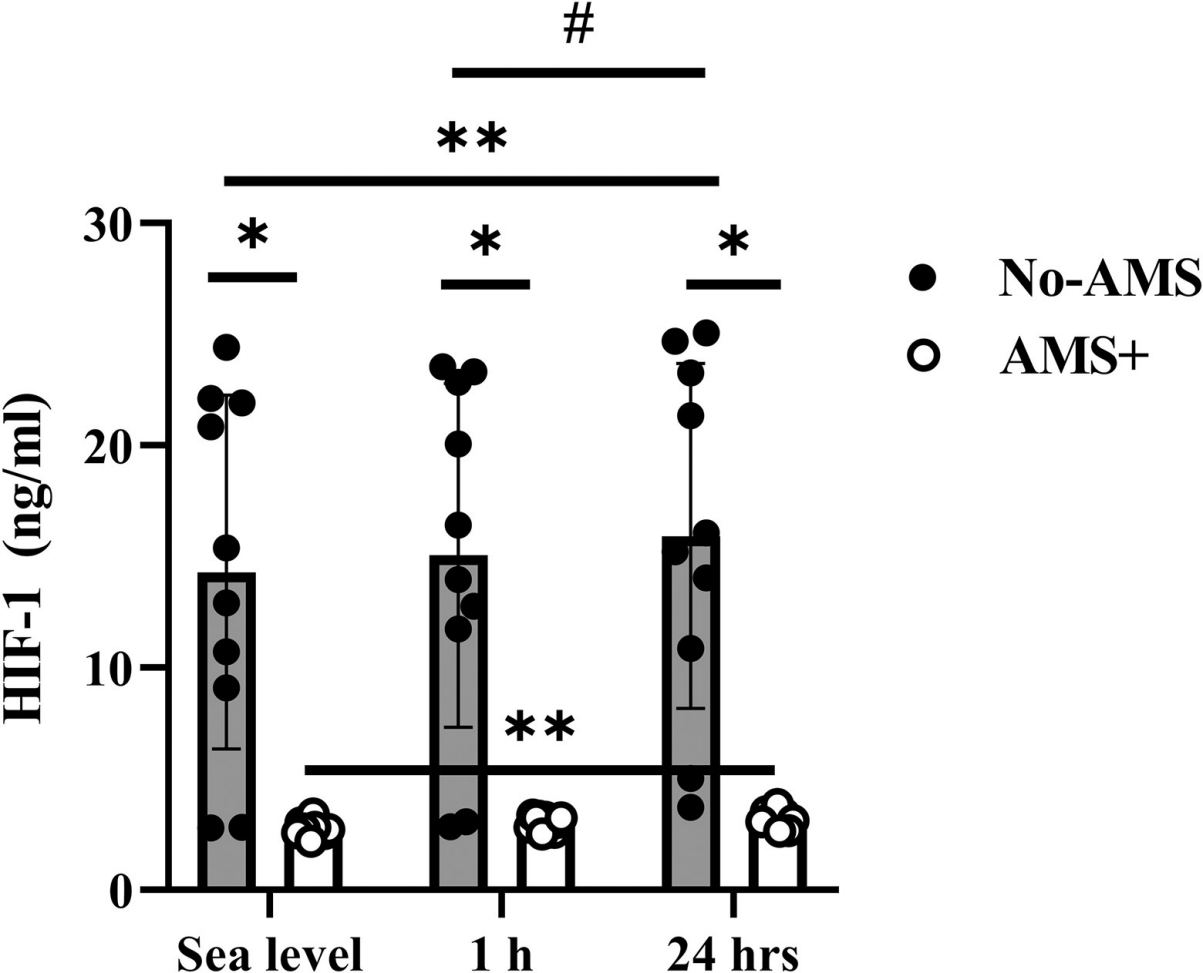
HIF-1 response to exposure to high altitude in No-AMS and AMS+ groups. * Significant difference between AMS and No-AMS groups, ** Significant different vs. Sea level, # Significant different vs. 1 h after exposure to high altitude. Sig≤0.05, values are Mean±SD.

As shown in Fig 2, HIF-1 increased 6% (P = 0.2) and 12% (P = 0.03) respectively at 1 and 24 h after exposure to altitude in compared to sea level in No-AMS group. Circulating HIF-1 rose 7% (P = 0.7) and 19% (P = 0.01) in 1 and 24 h after exposure to altitude in comparison sea level in AMS+ group. Furthermore, there was no significant difference between 1 and 24 h after exposure to high altitude in this group (P = 0.5).

A comparison between groups showed that the levels of HIF-1 at 1 (P = 0.0001) and 24 hrs (P = 0.0001) after exposure to high altitude in No-AMS group was more than AMS+ group (Fig 2).

#### Vascular Endothelial Growth Factor (VEGF)

**Fig 3:**
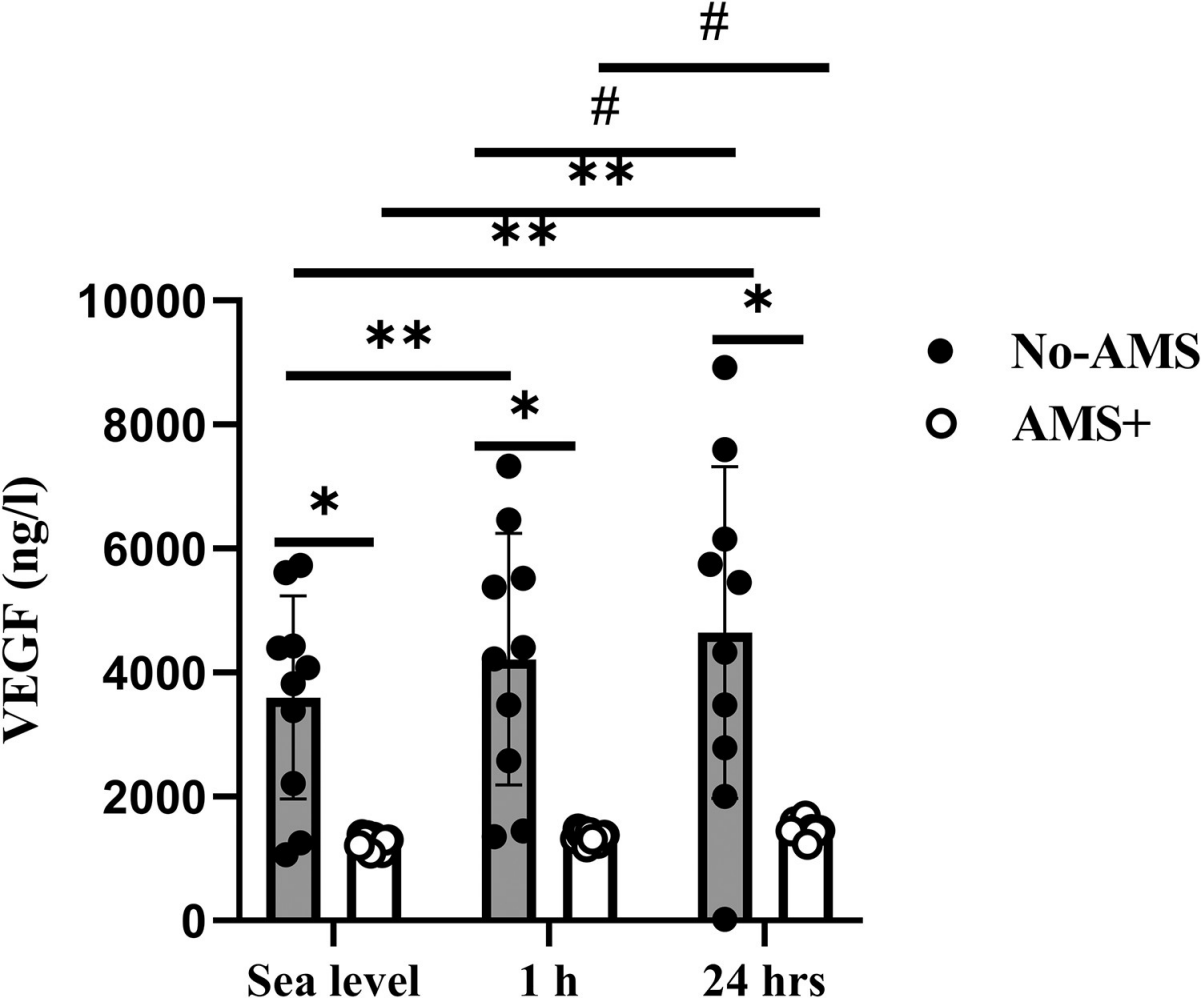
VEGF response to exposure to high altitude in No-AMS and AMS+ groups. * Significant difference between AMS and No-AMS groups, ** Significant different vs. Sea level, # Significant different vs. 1 h after exposure to high altitude. Sig≤0.05, values are Mean±SD.

VEGF increased significantly 1 and 24 h after exposure to high altitude in compared to sea level (respectively 17% and 34%) in No-AMS (Fig 3). Furthermore VEGF at 24 h after exposure to high altitude increased 15% in compared 1 h after exposure to high altitude (P = 0.01).

Serum VEGF increased in 1 and 24 h after exposure to high altitude compared to sea level (respectively, 7% (p = 0.07) and 28% (p = 0.01)) in AMS+ group. VEGF concentration in this group at 24 h after exposure to altitude was more in comparison to 1 h after exposure to high altitude (P = 0.01). At both time points of hypoxia, VEGF concentration was elevated in those who did not develop AMS as compared with those who did (respectively, P = 0.006 and P = 0.01).

#### Endothelin-1

**Fig 4:**
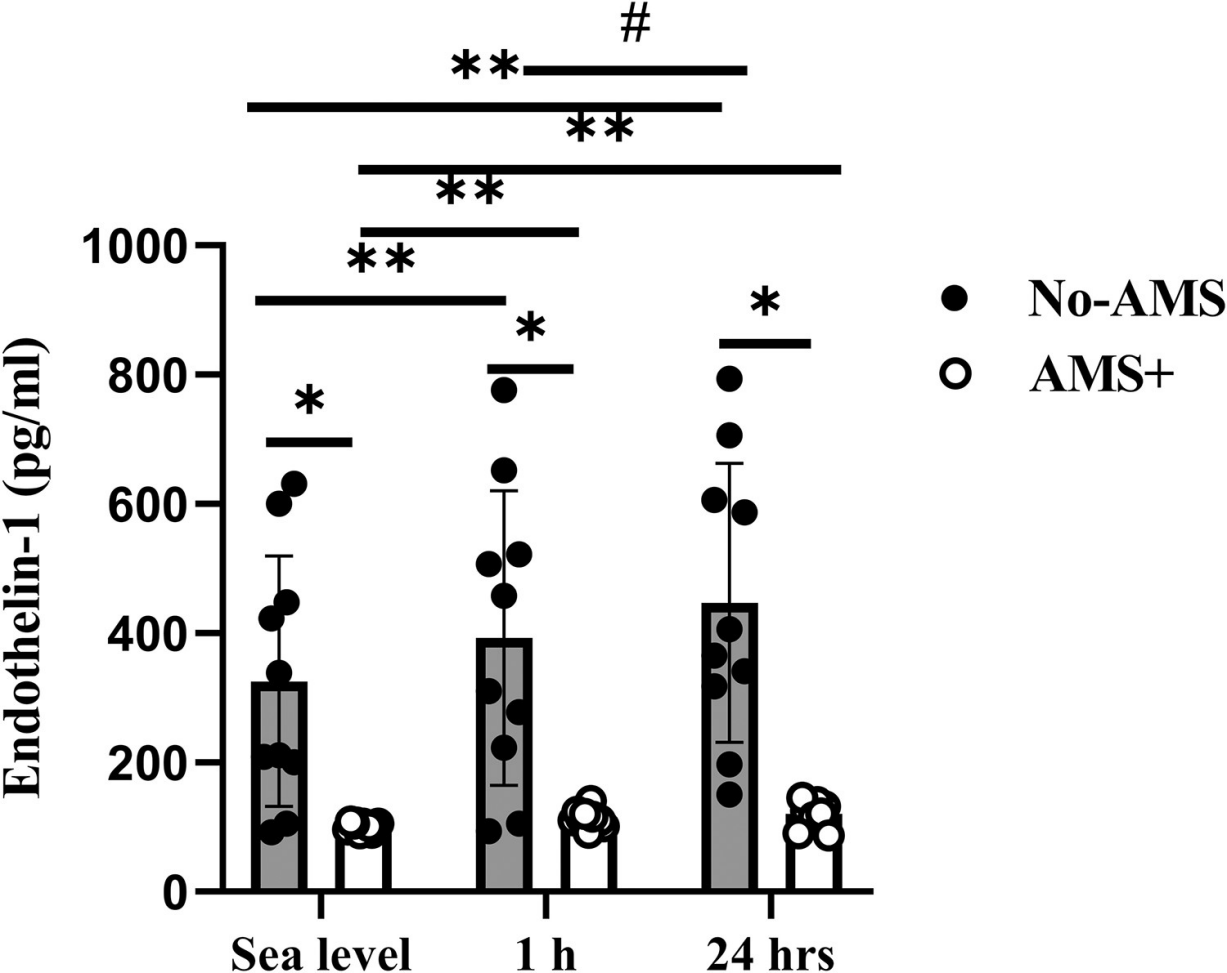
Endothelin-1 response to exposure to high altitude in No-AMS and AMS+ groups. * Significant difference between AMS and No-AMS groups, ** Significant different vs. Sea level, # Significant different vs. 1 h after exposure to high altitude. Sig≤0.05, values are Mean±SD.

Endothelin-1 increased 22% (P = 0.006) and 38% (P = 0.0001) at 1 and 24 h after exposure to altitude in compared to sea level in No-AMS group (Fig 4). Endothelin-1, 24 h after exposure to high altitude increased significantly in compared 1 h after exposure to altitude in this group(P = 0.03). Although there was a rise in Endothelin-1 at 1 h (10%) and 24 h (20%) after exposure to high altitude (respectively, P = 0.03 and P = 0.02) in AMS+ group, this was not different between time points (P = 1.0). Endothelin-1 concentration after exposure to hypoxia in AMS-resistant individuals was greater in compared to subjects who remained apparently healthy (P<0.05).

#### SpO_2_

**Fig 5:**
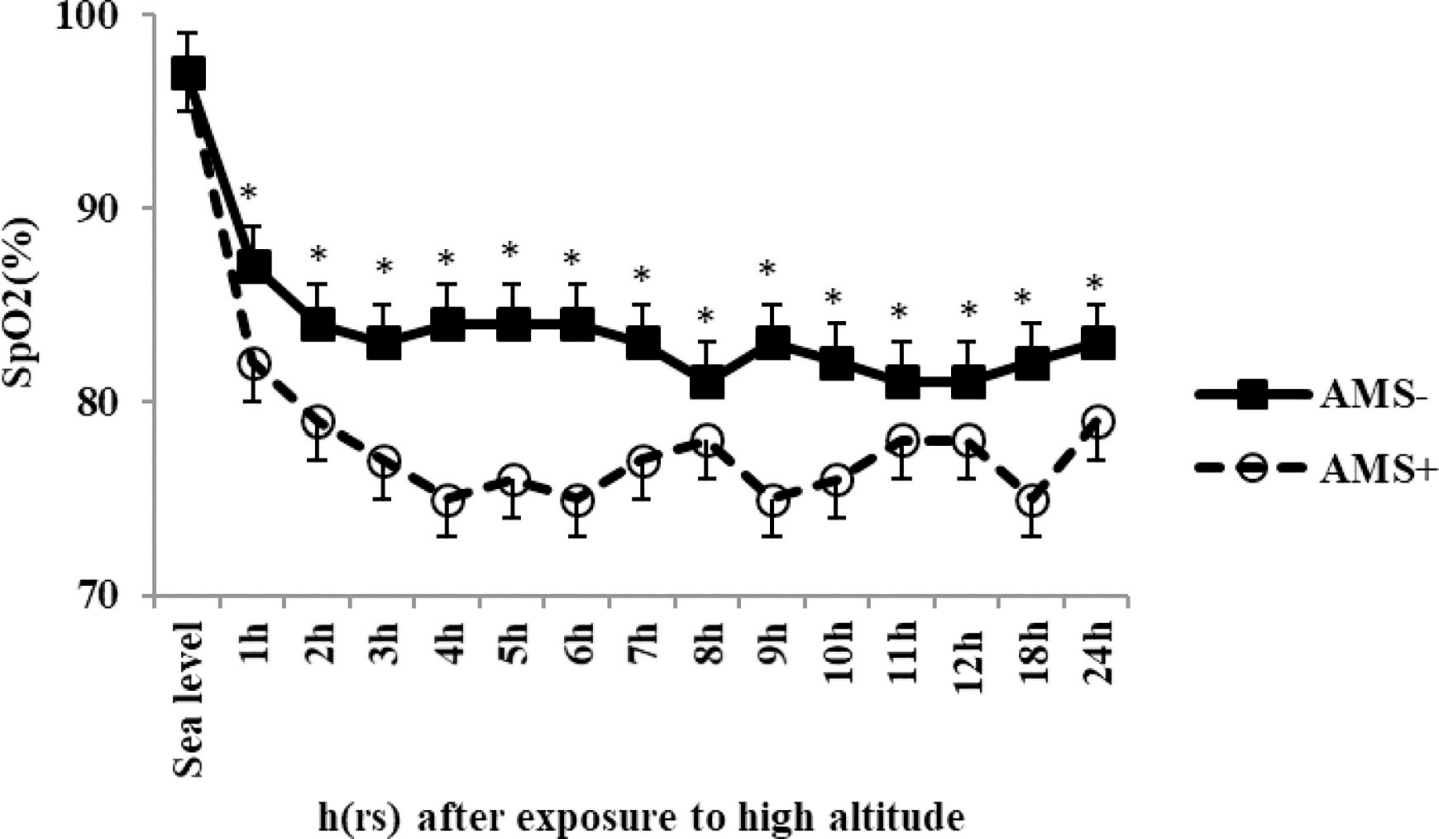
SpO_2_ changes in response to exposure to high altitude in No-AMS and AMS+, * show significant difference between AMS+ and No-AMS groups. Sig≤0.05, values are Mean±SD.

The mean of SpO_2_ during exposure to high altitude was 83.5±0.3 in No-AMS group and was 77.66±0.4 in AMS+ group. The mean of SpO_2_ decreased significantly 16% in No-AMS group and 25% in AMS+ group after exposure to altitude; this difference was significant (Fig 5). As well as, we found a significant difference for the mean of SpO_2_ between the AMS+ study group and the No-AMS study group (T = 10.8, P = 0.0001).

As shown in Table 3, at baseline, The Lake Louise score inversely correlate with Orexin-A, HIF-1, VEGF and Endothelin-1, as well as, no correlation was found between SpO_2_ at sea level and LLS. In 1 and 24 h after exposure to high altitude Lake Louise score was significant inversely correlation with Orexin-A, VEGF, HIF-1, Endothelin-1 and SpO_2_.

**Table 3:** The correlation between hypoxia related proteins with LLS at sea level and 1 and 24 hrs after exposure to high altitude.

*Factors*	Pearson Correlation	P value
*Sea level*		
Orexin-A	*-0*.*831*	*0*.*0001*
HIF-1	*-0*.*789*	*0*.*0001*
VEGF	*-0*.*774*	*0*.*0001*
Endothelin-1	*-0*.*691*	*0*.*001*
SpO_2_	*-0*.*181*	*0*.*43*
***1 h after exposure to altitude***		
Orexin-A	*-0*.*817*	*0*.*0001*
HIF-1	*-0*.*803*	*0*.*0001*
VEGF	*-0*.*769*	*0*.*0001*
Endothelin-1	*-0*.*710*	*0*.*0001*
SpO_2_	*-0*.*697*	*0*.*0001*
***24 h after exposure to altitude***		
Orexin-A	*-0*.*774*	*0*.*0001*
HIF-1	*-0*.*818*	*0*.*0001*
VEGF	*-0*.*753*	*0*.*0001*
Endothelin-1	*-0*.*770*	*0*.*0001*
SpO_2 mean_	*-0*.*884*	*0*.*0001*

## Discussion

The aim of the present study was to assess the potential role of the four hypoxia-related proteins in AMS pathogenesis. Taken together, the study’s main findings were that serum Orexin-A, HIF-1, VEGF and Endothelin-1 at sea level was more in AMS-resistant individuals, and all of them increased after exposure to altitude in No-AMS and AMS+; however, the magnitude of the Orexin-A response to the altitude at 1 and 24 h after exposure to hypoxia in individuals with AMS+ was approximately identical with No-AMS group. Elevation in HIF-1 24 hrs after exposure to altitude in AMS+ was more than No-AMS. In contrast, the increase in VEGF and Endothelin 1 and 24 hrs after exposure to altitude in No-AMS were more than AMS+.

In this study, difficulty sleeping and headache were the most common symptoms of individual with AMS. Meanwhile, Orexin-A has pivotal role in difficulty sleeping and sleep/wake regulation [12]. In support of our findings, Liu and colleagues confirmed that hypoxia increases the expression of Orexin-A and this change leads to sleep fragmentation after exposure to altitude [13]. Since Orexin-A to altitude was similar between groups, it is possible that other factors that regulate the sleep cycle are participate in difficulty sleeping after exposure to altitude in AMS+ group.

At sea level, 1 and 24 hrs after exposure to altitude, HIF-1 levels in AMS+ group was less than No-AMS group. In this regards, Kline and coworkers showed that HIF-1 partial deficient impaired ventilatory responses to chronic hypoxia [14]. The ventilatory response to the hypoxic is the main physiological response of the body at altitude, which can be directly related to adaptation to altitude and resistance to AMS. HIF-1 Elevations in HIF-1 24 hrs after exposure to high altitude in AMS+ was more compared to the No-AMS. It seems likely that HIF-responsive proteins such as VEGF and Endothelin-1 may predispose an individual to AMS. VEGF upregulated by hypoxia and demonstrated as an increase in vascular permeability [15]. There is an increasing body of evidence suggesting that VEGF is a potent permeability factor, although its role in altitude disease is obscure. In agreement with previous investigations, VEGF increased 1 and 24 h after exposure to hypoxia in AMS+ and No-AMS groups, but this change was more in No-AMS group (respectively 7% vs. 18% and 16% vs. 35%). In contrast of our findings Harrison et al. showed that serum VEGF at baseline was not different between AMS+ and No-AMS group; however, they did report that serum VEGF elevation was more in AMS+ group in comparison to No-AMS group at 3 days after exposure to altitude (43% vs. 26%) [16]. A probable explanation for the difference between the results of this study and the findings of Harrison related to the individual variability and different ascent profile, including the altitude at which measurements were made, duration of high altitude stay and the altitude of residency of the subjects. The mechanism behind VEGF elevation in response to hypoxia is unclear; however, it remains possible that less serum VEGF in subjects more susceptible to AMS may in part be due to enhanced renal VEGF clearance and fluid retention. For example, Maloney et al. showed that decrease in circulating VEGF after exposure to altitude may indicate protection against the development of VEGF-mediated capillary leak in organs such as the brain [17]. Furthermore, Kendall et al. showed that soluble receptors act to sequester unbound VEGF from the circulation, therefore preventing its interaction with the endothelium-bound receptors that mediate its effects upon endothelial function and vascular permeability [18]. While some studies have reported a relation between serum VEGF and AMS susceptibility, the majority did not [17, 19, 20]. It should to be noted that MRI brain scans following hypoxic exposure showed that brain swelling was not different between subjects who developed AMS and those who did not (An indirect argument against a role of VEGF in AMS) [19, 21]. Also, the concentration of VEGF in the cerebrospinal fluid is not different between subjects with and without AMS [22]. These findings demonstrated that vasogenic edema is not involved in the pathophysiology of AMS.

Previous experimental studies suggests that increased brain volume with hypobaric hypoxia elevates intracranial pressure and, when accompanied by impaired or diminished intracranial buffering capacity, contributes to the development of the symptoms that define AMS (tight-fit hypothesis). On the other hand, Julian and coworker for the first time showed that resistance to AMS may be driven, in part, by the ability to mount an adequate “defensive” anti-inflammatory or anti-permeability response after exposure to altitude [23]. These findings lend deep insight into the pathophysiology of AMS.

The increase of Endothelin-1 after exposure to hypoxia in both group are in line with previous studies [7, 24]. In contrast to these studies and our findings, Barker et al. showed that Endothelin-1 was not different between those with and without AMS susceptibility [6]. It was concluded that Endothelin -1 have a pathological role in HAPE instead of AMS [6].

The mean SpO_2_ of the AMS+ group after exposure to altitude was significantly lower than that of the No-AMS group. In contrast out findings Wagner et al. showed that there was no significant difference in the mean oxygen saturations at summit or average altitude between the AMS+ and No-AMS groups [25].

AMS symptoms do not appear immediately after exposure to altitude, it may last up to 12 hrs; therefore, if AMS can be predicted before it occurs, there will be enough time to prevent its possible adverse consequences. In fact, hypoxia related proteins (Orexin-A, HIF-1, VEGF and Endothelin-1) evaluation at sea level could be consider as a predictive indices to identify acute mountain sickness susceptibility individuals unaccustomed to high altitude. It should to be noted that the most important limitation of this study is that the sample size is not big enough.

## Conclusion

The results of this study in summery showed that hypoxia related proteins include Orexin-A, HIF-1, VEGF and Endothelin-1 were higher in the AMS-resistant individuals when compared to AMS susceptible individuals at sea level and these proteins did not show consistent changes after exposure to high altitude. Susceptibility to AMS may be associated with a high response HIF-1 and more SpO_2_ reduction to hypoxia. To better determine the underlying factors in AMS etiology, future work in this area should focus on genetic and local measurement of other factors. Further studies are needed to validate these measurements, but they suggest an alternative approach to obtain a more accurate simple method of predicting AMS.

## Supporting information

S1 Data ELISA data set of Orexin, HIF-1, VEGF and Endothelin-1. Abbreviation: AMS: Acute Mountain Sickness.
(XLS)
